# Machine learning based on the EEG and structural MRI can predict different stages of vascular cognitive impairment

**DOI:** 10.3389/fnagi.2024.1364808

**Published:** 2024-04-05

**Authors:** Zihao Li, Meini Wu, Changhao Yin, Zhenqi Wang, Jianhang Wang, Lingyu Chen, Weina Zhao

**Affiliations:** ^1^Department of Neurology, Hongqi Hospital Affiliated to Mudanjiang Medical University, Mudanjiang, China; ^2^Department of Neurology, Taizhou Second People’s Hospital, Taizhou, Zhejiang, China; ^3^Mudanjiang Medical College, Mudanjiang, China; ^4^Center for Mudanjiang North Medicine Resource Development and Application Collaborative Innovation, Mudanjiang, China

**Keywords:** machine learning, quantitative electroencephalogram, vascular cognitive dysfunction, structural magnetic resonance imaging, applications of support vector machine

## Abstract

**Background:**

Vascular cognitive impairment (VCI) is a major cause of cognitive impairment in the elderly and a co-factor in the development and progression of most neurodegenerative diseases. With the continuing development of neuroimaging, multiple markers can be combined to provide richer biological information, but little is known about their diagnostic value in VCI.

**Methods:**

A total of 83 subjects participated in our study, including 32 patients with vascular cognitive impairment with no dementia (VCIND), 21 patients with vascular dementia (VD), and 30 normal controls (NC). We utilized resting-state quantitative electroencephalography (qEEG) power spectra, structural magnetic resonance imaging (sMRI) for feature screening, and combined them with support vector machines to predict VCI patients at different disease stages.

**Results:**

The classification performance of sMRI outperformed qEEG when distinguishing VD from NC (AUC of 0.90 vs. 0,82), and sMRI also outperformed qEEG when distinguishing VD from VCIND (AUC of 0.8 vs. 0,0.64), but both underperformed when distinguishing VCIND from NC (AUC of 0.58 vs. 0.56). In contrast, the joint model based on qEEG and sMRI features showed relatively good classification accuracy (AUC of 0.72) to discriminate VCIND from NC, higher than that of either qEEG or sMRI alone.

**Conclusion:**

Patients at varying stages of VCI exhibit diverse levels of brain structure and neurophysiological abnormalities. EEG serves as an affordable and convenient diagnostic means to differentiate between different VCI stages. A machine learning model that utilizes EEG and sMRI as composite markers is highly valuable in distinguishing diverse VCI stages and in individually tailoring the diagnosis.

## 1 Introduction

Vascular cognitive impairment (VCI) is a leading cause of chronic progressive cognitive impairment in the elderly population and is caused by cerebrovascular lesions and their associated risk factors (Biesbroek and Biessels, 2023). Vascular cognitive impairment (VCI) spans a spectrum including subjective cognitive decline (SCD), vascular cognitive impairment with no dementia (VCIND), and vascular dementia (VD). Today’s research has shown that in people with vascular cognitive impairment (VCI), many subtle changes in the structure and function of the brain have taken place prior to the appearance of overt cognitive impairment and clinical deficits (Sang et al., 2020; Yang et al., 2022; Badji et al., 2023). Some of the most significant challenges at present are to identify brain disorders that show VCI in the early stages of the disease and, if possible, to identify those that may progress to VD. However, to date, the structural brain characteristics and electrophysiologic functional changes in different stages of VCI have not been quantitatively distinguished in any relevant study. Mechanical learning methods that combine neuroimaging features have been utilized in recent years for early VCI diagnosis, demonstrating significant potential (Lu et al., 2020; Li et al., 2021). However, there has been relatively little study on combined neuroimaging and neurophysiology. Due to the limitations of unimodal studies, a combined multimodal analysis that incorporates both neuroimaging and neurophysiology may offer a novel approach for identifying the structural and functional changes in the brains of VCI patients at different stages. This could potentially serve as a biomarker for identifying the various stages of VCI and pave the way to explore new therapeutic targets. This study aimed at investigating precision of sMRI and resting-state EEG in discriminating between different stages of VCI, and at integrating both techniques in discriminating between VCI, VCIND, and healthy elderly using a support vector machine classification.

## 2 Materials and methods

**FIGURE 1:**
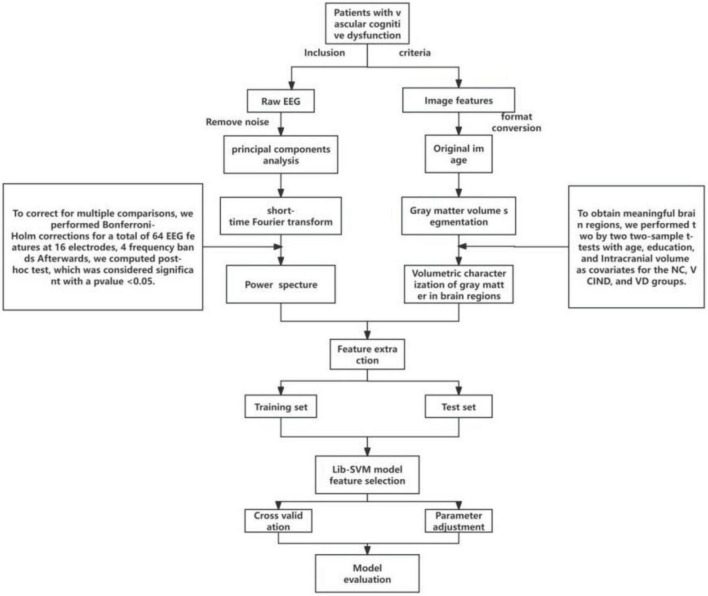
Data processing flow chart.

All participants with VCI in the study were patients who visited the Memory Clinic and the Ward of the Department of Neurology at Hongqi Hospital, Affiliated to Mudanjiang Medical College, from September 2021 to October 2022, with the primary complaint of memory loss. All participants without cognitive impairment were recruited from the general community or from physical examinations at memory clinics. All participants provided informed consent prior to their inclusion in the study. The detailed methodologies are described below and in Figure 1. The study was conducted in adherence with the guidelines laid out in the Declaration of Helsinki. The Ethical Review Committee of Hongqi Hospital, affiliated with Mudanjiang Medical College, approved the study (Ethics No. 2022011).

### 2.1 Inclusion and exclusion criteria

All participants were sorted into three groups after undergoing the Montreal Cognitive Assessment (MoCA) and the clinical dementia rating scale (CDR) with physician supervision. These groups consist of the NC group (*n* = 30), the VCIND group (*n* = 32), and the VD group (*n* = 21). For the Montreal Cognitive Assessment (MoCA) (Nasreddine et al., 2005), a cutoff value of < 26 indicates cognitive impairment. Additionally, one point is added to the raw MoCA score if the participant’s education level is < 12. All participants in the study were right-handed individuals of Han Chinese descent from northeastern China. Enrollment requirements for the NC group included: (1) absence of cognitive decline complaints; (2) a MoCA score of 26 or higher and a CDR score of 0; (3) no identification of symptoms or positive signs during the physical examination, and (4) no significant anomalies found in the head magnetic resonance imaging. Enrollment requirements for the VCIND group included (Sachdev et al., 2014): (1) a complaint or evidence of cognitive dysfunction from a knowledgeable source, with normal or slightly impaired ability to perform daily activities; (2) a MoCA score less than 26 and a CDR score equal to 0 or 0.5; and (3) an intracranial lesion visible on imaging, meeting VCI diagnostic criteria and clearly linked to cognitive decline. Enrollment requirements for the VD group included (Erkinjuntti, 1994): (1) significant cognitive impairment, reported by the patient or by an appropriate caregiver, affecting activities of daily living, (2) MoCA score < 22 and CDR score ≥ 1, and (3) cognitive impairment from VD confirmed by imaging evidence of intracranial pathology meeting diagnostic criteria and clearly associated with patient cognitive impairment. Exclusion criteria: (1) The participant has a history of heart or kidney disease, cancer, or other significant systemic illness. (2) The cognitive decline is unrelated to cerebrovascular disease. (3) The participant experiences progressive memory or cognitive decline without associated imaging changes. (4) The participant is unable to communicate due to severe impairment in hearing, vision, or speech; (5) The participant displays symptoms of depression and anxiety according to the Hamilton depression and anxiety inventory; (6) The participant has a history of mental illness or congenital developmental abnormalities; (7) The participant refuses or is unable to undergo brain MRI and EEG; (8) The participant experienced an acute cerebral infarction within the past three months.

### 2.2 Neuropsychological tests

All participants underwent a thorough neuropsychological evaluation, which assessed their verbal and visual situational memory, attention, executive function, visuospatial skills, and language proficiency. The evaluation utilized various specific tests, such as the Montreal Cognitive Assessment (MoCA), shape trails test (STT), shape trails test-A (STT-A), and shape trails test-B (STT-B). The STT consisted of two components: shape trails test-A (STT-A) and shape trails test-B (STT-B). All neuropsychological assessments were performed under the guidance of a specialist physician in neurology.

### 2.3 Acquisition of EEG data

Electroencephalography (EEG) data were collected early in the morning, between 8:00 and 9:30 a.m., with patients awake, fasted, and in a quiet, closed-eye state. All EEG data were recorded on the same digital EEG system (NicoletOne™ EEG system, Natus Neurology Inc.), and a uniform signal acquisition standard was used to eliminate bias caused by different EEG equipment and parameters. Standardization of signal acquisition was used to eliminate bias caused by different EEG equipment and parameters. We collected data by placing a total of 16 electrodes (including Fp1, Fp2, F7, F8, F3, F4, C3, C4, T3, T4, T5, T6, P3, P4, O1, and O2) according to the international 10–20 standard lead system at a sampling rate of 250 Hz, with the input impedance set to Z > 100 MΩ, and collected the EEG signals for at least 30 min.

### 2.4 EEG data processing

(1) We used the EEGLAB toolkit^1^ based on matlab2019b^2^ to localize electrodes, reject useless electrodes, and perform mean-based data re-referencing for all EEG data. (2) Select low frequency 1HZ high frequency 30HZ to filter and save the file; (3) Two or more EEG experts visually analyze the data and remove bad segments and artifacts; (4) Run the EEGLAB independent component analysis (ICA) toolbox to analyze the data for principal components and remove ICA-unusable components; (5) Extraction of EEG power spectra in each frequency band based on short-time Fourier transform. (6) Finally, 64 qEEG features (16 channels, 4 frequency bands) were extracted for each patient, and we performed multivariate analysis of covariance (MANCOVA) with age, sex, and education as covariates for the NC, VCIND, and VD groups, followed by multiple comparisons to control for error rates at the level of statistical significance (using Bonferroni-Holm correction), and after Bonferroni-Holm correction, *post-hoc* tests were performed and significance was determined at *p* < 0.05.

### 2.5 Nuclear magnetic resonance data acquisition

Magnetic resonance imaging (MRI) data acquisition for all subjects was performed on a Philips Achieva 3.0T MRI machine, using an 8-channel head coil, performing routine cranial transverse T1WI sequence scans. Scanning parameters: FOV = 256 mm × 256 mm^2^, slice thickness = 1 mm, GAP = 0, number of slices = 192, TR/TE/TI = 7/3.2/1,100 ms, 7° flip angle, matrix = 256 × 256.

### 2.6 Magnetic resonance data processing

We used MRIcron, SPM12 and DPABI software package to analyze the NMR data and calculate the gray matter volume of the whole brain voxel. All of the above were run on MatLab (R2019b). The main steps were as follows: (1) MRIcron software was used to convert the MRI data DICOM files of all participants into NIfTI files; (2) the NIfTI files were imported into CAT12 in SPM12 for segmentation; (3) quality checks were performed, and the segmented gray matter image was smoothed; (4) the smoothed data were imported into DPABI for statistical analysis and image presentation (all gray matter structures were partitioned using Anatomical Automatic Labeling). (5) Using DPABI, the significant brain regions obtained from the Voxel-based morphometry (VBM) analysis were set as regions of interest (ROI), and the gray matter volumes of the ROI were obtained.

### 2.7 Machine learning feature filtering

In this study, a support vector machine (SVM) model is constructed and the algorithm consists of two main steps: training of the SVM classifier and evaluation of the model. We divided the 83 samples into a training set and a test set at a ratio of 8:2 to ensure the generalization performance of the model. In the model of the NC group with the VCIND group, 24 NC participants and 26 patients of the VCIND group were randomly selected as the training set to build the SVM model, and the remaining 12 were used as the test set; in the NC group and VD group model, 24 NC participants and 17 VD group patients were selected at random as the training set to build the SVM model, and the remaining 10 were used as the test set; and in the VCIND group and VD group model, 26 VCIND patients and 17 VD patients were selected at random as the training set, and the remaining 10 patients were used as the test set. In this study, we performed quantitative electroencephalogram (qEEG) power spectrum analysis and VBM analysis on the test sets of the NC group vs. VCIND group, the NC group vs. VD group, and the VCIND group vs. VD group, and we selected statistically significant (*p* < 0.05) data obtained from two-way comparisons as categorical features (qEEG power spectra and volume of brain area corresponding to gray matter atrophy).

Then the LibSVM toolbox^3^ in MATLAB is used for support vector machine classification, the model has two key parameters: the kernel function and the regularization parameter, to optimize the model’s performance, we chose the radial basis function as the kernel function and used the grid search method in quintuple cross-validation to determine the regularization parameter. We used the model on the training set to predict the diagnostic results on the test set and evaluated the predictive ability of the model using the receiver operating characteristic (ROC) curve and the area under the ROC curve (AUC-ROC). This approach helps to prevent overfitting of the model on the training set and thus allows for a more accurate assessment of the generalization ability of the model.

### 2.8 Data analysis

SPSS version 21 was used to analyze all clinical and demographic data between groups. Count data are presented as case numbers (proportion), and Fisher’s exact test was used to analyze participant demographics. Normally distributed data are presented as mean ± standard deviation, whereas non-normally distributed data are presented as M (Q1, Q3). Normally distributed data were analyzed by one-way analysis of variance (ANOVA), and the least significant difference was used for *post-hoc* testing. Non-normally distributed data were tested using the Kruskal-Wallis H test, an independent-samples non-parametric test (*p* < 0.05 was regarded as statistically significant). All MRI image data were analyzed using DPABI. Age, sex, education level, and total brain volume were used as covariates, and the permutation test was used to correct for multiple comparisons to analyze gray matter atrophy changes in participants.

## 3 Results

### 3.1 Demographic and clinical characteristics

The study included 83 subjects, and Table 1 displays their demographic characteristics. The three patient groups differed significantly in age, education level, history of hypertension, and level of cognitive impairment (*P* < 0.05).

**TABLE 1:** Sample demographic and clinical characteristic.

	NC (*n* = 31)	VCIND (*n* = 32)	VD (*n* = 21)	F	*P*
Sex (male)%	60.1	60.7	53	\	0.089
Age	54.84 ± 7.82	59.41 ± 7.35	60.67 ± 10.93	3.586	0.032*
Education	10.53 ± 3.35	8.91 ± 3.55	7.24 ± 3.37	5.829	< 0.01**
Hypertension	5	21	13	20.232	< 0.01**
Coronary heart disease	2	3	2	0.092	0.912
Atrial fibrillation	2	0	0	0.056	0.946
Diabetes	8	11	6	2.311	0.106
Valvular heart disease	0	2	2	0.659	0.520
Smoking	12	10	6	0.690	0.505
MoCA	26 ± 2.4	22 ± 1.3	18.5 ± 4.4	\	< 0.01**
STT-A	45.6 ± 27.1	132.5 ± 3.3	181.7 ± 90.05		0.028*
STT-B	76.1 ± 38.4	157 ± 2.0	200.5 ± 2.2	\	< 0.01**

NC, healthy controls; VCIND, vascular cognitive impairment with no dementia; VD, vascular dementia;

*p < 0.05;

**p < 0.01.

### 3.2 Results of sMRI

**FIGURE 2:**
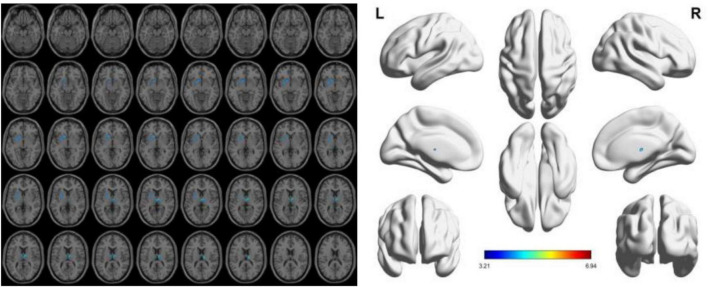
The presence of gray matter atrophy in the VCIDS group compared to the NC group, indicated in blue, with a statistically significant difference (*p* < 0.05).

**FIGURE 3:**
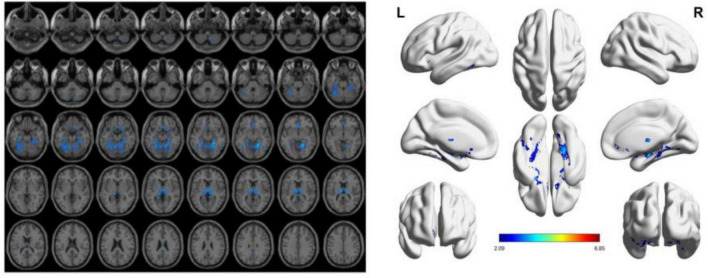
The presence of gray matter atrophy in the VD group compared to the VCIND group, indicated in blue, with a statistically significant difference (*p* < 0.05).

**FIGURE 4:**
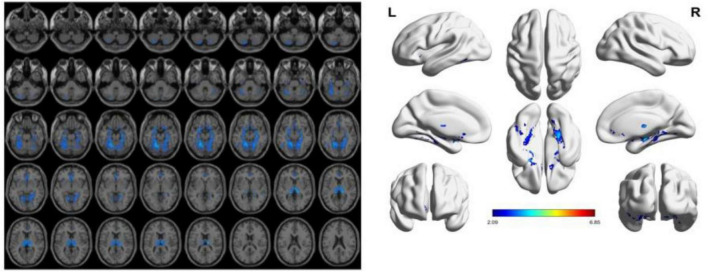
The presence of gray matter atrophy in the VD group compared to the NC group, indicated in blue, with a statistically significant difference (*p* < 0.05).

To reduce confounding, we used patient age, sex, education, and intracranial volume as covariates and corrected for multiple comparisons using the permutation test to analyze changes in gray matter atrophy between participants. In our study, VBM analysis showed significant differences only in gray matter in the Putamen_L, Caudate_L, and Thalamus_R regions when comparing the NC group with the VCIND group (as shown in Figure 2 and Table 2), but differences in more extensive gray matter atrophy were seen when comparing the VCIND group with the VD group and the NC group with the VD group. In the comparison between the VCIND group and the VD group, it showed atrophy in 18 relevant brain regions including Fusiform_L, Cerebelum_6_L, Cerebelum_4_5_R, Fusiform_R, Lingual_R, Cerebelum_4_5_L, Cerebelum_6_R, and so on (as shown in Figure 3 and Table 2); while the comparison between NC and VD groups showed atrophy of 25 brain regions including Thalamus_L, Thalamus_R, Fusiform_L, Olfactory_L, Cerebelum_6_L, Cerebelum_4_5_R, Fusiform_R, and Lingual_R (as shown in Figure 4 and Table 2).

**TABLE 2:** Feature extraction and selection.

	NC (*n* = 31)	VCIND (*n* = 32)	VD (*n* = 21)	*p*
Intracranial volume	1,319.24 ± 136.50	1,331.75 ± 169.48	1,358.54 ± 151.86	0.667
Gray matter volume	549.41 ± 42.43	533.39 ± 54.83	494.82 ± 58.84	0.002**
White matter volume	470.39 ± 44.78	473.00 ± 62.44	428.33 ± 70.52	0.017*
Cerebelum_Crus2_L^b^^c^	2.70 ± 0.76	2.82 ± 0.76	2.37 ± 0.96	0.133
Cerebelum_8_L^c^	3.11 ± 1.03	3.18 ± 0.90	2.53 ± 1.07	0.051
Cerebelum_6_L^b^^c^	4.80 ± 0.48	4.71 ± 0.61	4.36 ± 0.63	0.023*
Fusiform_L^b^^c^	4.39 ± 0.43	4.24 ± 0.51	3.86 ± 0.52	0.001**
Thalamus_R^a^^b^^c^	5.55 ± 0.47	5.57 ± 0.78	4.50 ± 0.82	0.000**
Thalamus_L^b^^c^	5.24 ± 0.40	5.30 ± 0.77	4.46 ± 0.95	0.000**
Cerebelum_6_R^b^^c^	4.60 ± 0.57	4.56 ± 0.62	4.10 ± 0.57	0.007**
Lingual_R^b^^c^	3.26 ± 0.36	3.22 ± 0.34	2.98 ± 0.37	0.013*
Cerebelum_4_5_R^b^^c^	4.74 ± 0.58	4.59 ± 0.71	4.39 ± 0.67	0.179
Cerebelum_4_5_L^b^^c^	4.26 ± 0.48	4.18 ± 0.63	3.91 ± 0.60	0.089
Fusiform_R^b^^c^	4.37 ± 0.54	4.26 ± 0.48	3.95 ± 0.59	0.021*
Lingual_L^b^^c^	3.41 ± 0.33	3.35 ± 0.34	3.16 ± 0.45	0.053
ParaHippocampal_R^b^^c^	4.43 ± 0.49	4.33 ± 0.46	4.03 ± 0.50	0.015*
Hippocampus_R^c^	4.97 ± 0.46	4.94 ± 0.49	4.30 ± 0.56	0.000**
Hippocampus_L^c^	5.10 ± 0.50	5.00 ± 0.56	4.40 ± 0.64	0.000**
Cingulum_Ant_L^b^^c^	3.82 ± 0.48	3.74 ± 0.51	3.47 ± 0.60	0.062
Olfactory_L^c^	5.43 ± 0.74	5.26 ± 0.69	4.83 ± 0.85	0.022*
Temporal_Inf_L^b^^c^	3.84 ± 0.39	3.81 ± 0.43	3.41 ± 0.58	0.002**
ParaHippocampal_L^b^^c^	4.11 ± 0.40	4.07 ± 0.41	3.87 ± 0.52	0.123
Amygdala_R^c^	5.74 ± 0.58	5.67 ± 0.61	5.16 ± 0.69	0.003**
Amygdala_L^c^	6.39 ± 0.67	6.29 ± 0.71	5.66 ± 0.88	0.002**
Rectus_R^b^^c^	4.26 ± 0.55	4.21 ± 0.55	3.86 ± 0.59	0.032*
Olfactory_R^b^^c^	5.49 ± 0.68	5.37 ± 0.69	4.99 ± 0.89	0.057
Vermis_4_5^b^^c^	3.36 ± 0.37	3.12 ± 0.44	3.11 ± 0.44	0.036*
Insula_L^c^	4.76 ± 0.53	4.72 ± 0.50	4.27 ± 0.69	0.006**
Putamen_L^a^	5.89 ± 0.65	5.94 ± 0.69	5.39 ± 1.12	0.038*
Caudate_L^a^	4.91 ± 0.70	5.06 ± 0.93	4.63 ± 1.30	0.288
Theta^abc^	0.23 ± 0.02	0.18 ± 0.02	0.31 ± 0.0	< 0.01**
Delta^b^^c^	0.41 ± 0.05	0.14 ± 0.03	0.26 ± 0.17	0.020*
Alpha2^c^	0.20 ± 0.01	0.21 ± 0.17	0.21 ± 0.29	0.023*
Theta/gamma^b^^c^	0.14 ± 0.12	0.24 ± 0.05	0.17 ± 0.10	0.044*
Alpha1/Alpha2^a^^c^	0.37 ± 0.28	0.17 ± 0.21	0.22 ± 0.17	0.032*
Beta1^a^^c^	0.23 ± 0.14	0.20 ± 0.08	0.30 ± 0.07	0.018*
Beta2^b^^c^	0.31 ± 0.22	0.11 ± 0.22	0.28 ± 0.12	0.007*

^a^Denotes relevant brain regions with significant gray matter atrophy or power spectra with significant differences in the comparison between the NC and VCIND groups.

^b^Denotes relevant brain regions with significant gray matter atrophy or power spectra with significant differences in the comparison between the VCIND and VD groups.

^c^Denotes relevant brain regions with significant gray matter atrophy or power spectra with significant differences in the comparison between the NC and VD groups.

*p < 0.05;

**p < 0.01.

### 3.3 EEG analysis results

**FIGURE 5:**
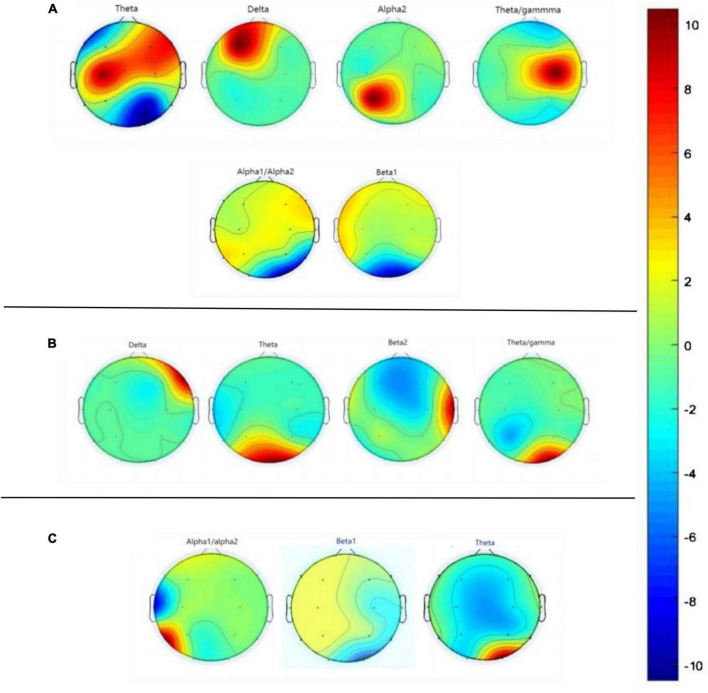
Graph **(A)** represents the difference in EEG power spectra between NC and VD groups, graph **(B)** represents the difference in EEG power spectra between VD and VCIND groups, and graph **(C)** labels the difference in EEG power spectra between VCIND and NC groups.

In our experiment, the VCIND group exhibited an increase in theta power in the O2 region and a decrease in beta power in the O1-O2 region compared to the NC group. Three EEG power spectra were statistically significant: the T3-O1 (Alpha1/Alpha2), O2 (Beta1), and O2 (Theta) waves. As for the comparison between the VD and VCIND groups, in addition to the power changes in the O1-O2 region, it also showed an increase in theta in the frontal and parietal lobes and a higher delta power in the F3-F4 region, where the theta difference was most pronounced, and there were a total of four power spectral features with statistically significant differences, namely F4 (delta), O1-O2 (theta), T4 (beta2), and O2 (theta/gamma);The comparison of the NC and VD groups revealed significant differences in six power spectra: P3 (Theta), F3 (Delta), O1 (Alpha2), P4 (Theta/gamma), O2 (Alpha1/Alpha2), and O1-O2 (Beta1). Refer to Figure 5 and Table 2 for more details.

### 3.4 Feature extraction and selection

Based on the results of the VBM analysis, we labeled voxels suggesting statistical significance, calculated the volume of brain regions in their areas, and analyzed them (see Table 2 for details). We believe that since some features are useless, irrelevant or redundant for classification, too many features can lead to “overfitting,” so eliminating useless features and selecting meaningful ones not only simplifies the classification model, but also improves the classification accuracy. In the training set, we selected 3 power spectral features and 2 sMRI features that were statistically significant in the NC group compared with the VCIND group (see Table 2 for details, the upper right corner is labeled as a); 4 power spectral features and 11 sMRI features that were statistically significant in the VCIND group compared with the VD group (see Table 2 for details, the upper right corner is labeled as b); 6 power spectral features and 16 sMRI features that were statistically significant in the NC group compared with the VD group (see Table 2 for details, the upper right corner is labeled as c).

### 3.5 Machine learning models

**FIGURE 6:**
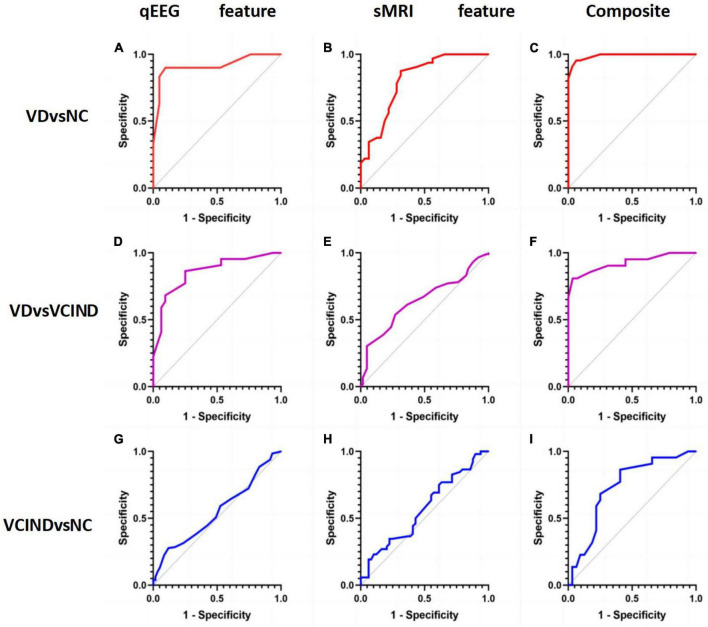
**(A)** shows the accuracy of distinguishing VD group from NC group by qEEG feature model in support vector machine machine learning model; **(B)** shows the accuracy of distinguishing VD group from NC group by sMRI feature model in support vector machine machine learning model; **(C)** shows the accuracy of distinguishing VD group from NC group by composite markers to distinguish the accuracy of VD group from NC group; **(D)** shows the accuracy of distinguishing VD group from VCIND group by qEEG feature model in support vector machine machine learning model; **(E)** shows the accuracy of distinguishing VD group from VCIND group by sMRI feature model in the support vector machine machine learning model; **(F)** shows the accuracy of distinguishing VD group from VCIND group by composite markers in the support vector machine machine learning model; **(G)** shows the accuracy of distinguishing VCIND group from NC group by qEEG feature model in the support vector machine machine learning model; **(H)** shows the accuracy of distinguishing VCIND group from NC group by sMRI feature model in the support vector machine machine learning model. **(H)** represents the accuracy of distinguishing VCIND group from NC group by sMRI feature model in the machine learning model of support vector machine. **(I)** shows the accuracy of distinguishing VCIND group from NC group by composite markers in the support vector machine machine learning model.

Our study shows that sMRI has better classification ability than qEEG in distinguishing VD from cognitively normal people. The area under the ROC curve of the sMRI-based support vector machine learning model is AUC = 0.90, and the area under the ROC curve of the machine learning classification model based on the qEEG features is AUC = 0.82. The “composite marker” model that combines sMRI and qEEG achieves the best classification results, with an area under the ROC curve of AUC = 0.98 (Table 3 and Figures 6A–C). When distinguishing between VD and VCIND populations, sMRI demonstrated better classification ability than qEEG. The machine learning model based on sMRI had an area under the ROC curve of AUC = 0.80, while the machine learning classification model based on qEEG features had an AUC of only 0.64. The composite marker model, which combined sMRI and qEEG, achieved optimal classification results with an ROC curve AUC of 0.92 (Table 3 and Figures 6D–F). When using only sMRI or qEEG features to differentiate between VCIND patients and NC, both methods had poor classification ability, with an area under the ROC curve of 0.56 for sMRI features and 0.54 for qEEG features. However, the “composite marker” model, which combines both sMRI and qEEG features, achieved relatively good classification ability with an area under the ROC curve of 0.72 (Table 3 and Figures 6G–I).

**TABLE 3:** Projected results.

	Feature	Accuracy%	Sensitivity%	Specificity%	AUC
VD vs. HC	sMRI	82.10	83.33	77.34	0.9
	qEEG	64.28	58.21	71.28	0.82
	sMRI+qEEG	86.28	88.31	79.79	0.98
VD vs. VCIND	sMRI	62.31	60.23	68.36	0.8
	qEEG	60.14	57.28	61.24	0.64
	sMRI+qEEG	84.10	82.35	74.38	0.92
VCIND vs. HC	sMRI	54.10	53.18	55.85	0.56
	qEEG	51.03	50.64	53.18	0.54
	sMRI+qEEG	73.85	69.24	70.01	0.72

NC, healthy controls; VCIND, vascular cognitive impairment with no dementia; VD, vascular dementia.

## 4 Discussion

This study applies a machine learning method that combines sMRI with qEEG to compare the classification ability of single-mode markers of qEEG or sMRI and composite markers of qEEG+sMRI for VCIND and VD. We found that EEG performed well in differentiating between VD and NC, with an AUC score of 0.82. One of the strongest predictors was elevated theta power, and this effect was similarly demonstrated in several regions, such as P3, O1, and O2 electrodes. In contrast, the EEG model exhibited lower accuracy in classifying the VCIND group compared to the NC group, with an AUC score of only 0.54. The optimal EEG features for classification differed from those used to differentiate between VD-NC, mainly in the form of an increase in theta power at the O2 electrode and a decrease in Beta power in the O1-O2 region, as was also found in a previous study (Babiloni et al., 2021). Throughout history, fluctuations in theta power have been associated with learning and memory (Herweg et al., 2020). Theta power has been linked to the exchange of information between hippocampus, entorhinal, perirhinal, and parahippocampal cortices and the memory of constituent events (Mayes et al., 2007). Previous studies have confirmed that an increase in theta power correlates with the severity of vascular injury (Herweg and Kahana, 2018). Additionally, a decrease in beta power has been found to correlate with dementia (Giustiniani et al., 2023). The neurophysiologic changes associated with VCI are primarily characterized by damage to the neurovascular unit (NVU). The neurovascular unit (NVU) is a complex anatomical structure composed of blood-brain barrier-specialized endothelial cells surrounded by the basal lamina and interacting with neurons, astrocytes, microglia, pericytes, and extracellular matrix (Iadecola, 2017; Zanon Zotin et al., 2021; Rundek et al., 2022). Damage to the NVU in the early pathogenic stages may lead to impaired regulation of cerebral blood flow, vascular permeability, immune transport, and waste removal. Reduced perfusion flow to brain tissue and vascular risk factors, such as hyperlipidemia, hyperglycemia, and hyperuricemia, due to intracranial atherosclerosis, stenosis, and occlusion, have superimposed effects that significantly increase the production of pro-inflammatory molecules and cytokines. This leads to increased neuroinflammation, damage to axons, and consequent slowing of neural conduction, ultimately resulting in altered rhythms of electrophysiological activity in the brain (Torres-Simón et al., 2022). Previous studies have demonstrated that an increase in slow-wave activity (delta and theta) and a decrease in fast-wave activity (alpha and beta) reflects the loss of synaptic innervation during the progression of the disease (Musaeus et al., 2018). Our experiments have yielded similar results. Theta power is widely regarded as the most reliable predictor of patient status. An increase in theta is one of the earliest neurophysiological changes observed in mild cognitive impairment (Chino-Vilca et al., 2022). In our study, the most significant difference in theta was observed between the VCIND group and the NC in the posterior head region, specifically O1-O2. In addition to the posterior head region, the VD group with the VCIND group also showed increased theta power in the frontal and parietal lobes and higher delta power in the F3-F4 region. These changes may reflect broader cerebral cortex changes during the later stages of VCI. The delta power changes occur at a later stage.

In the present study, we also evaluated the early predictive value of qEEG and sMRI in patients with VCI. In the qEEG study, we found that increased theta power in the posterior head showed the best results in differentiating the VD-NC group, suggesting that theta power may be an early clinical manifestation of neurodegeneration, and Chen et al. (2008) also concluded that changes in theta power are associated with dysfunction of brain networks, and that the elevation of theta power in fMRI in corresponding brain regions is inversely proportional to the BOLD signal (Laufs, 2008), which further supports our view. In addition, animal studies have shown that theta waves are generated in the hippocampus and are associated with functional changes in the hippocampus (Fox et al., 1986). Accordingly, we propose that changes in theta power in the early stages of vascular cognitive impairment may be a marker of hippocampal impairment and disruption of functional brain network connectivity in patients with VCI. Cognitive impairment in VCI has long been reported in previous studies (Hajjar et al., 2015; Boomsma et al., 2022), mainly including executive function (Hajjar et al., 2009; Degen et al., 2016), visuospatial function (Degen et al., 2016), and situational memory (Song et al., 2020), and these cognitive alterations are inextricably linked to structural changes in the brain of VCI patients. In our experiments, the sMRI model outperformed EEG in distinguishing VD from NC and VCIND participants. Both achieved high classification accuracy with VD vs. HC: AUC = 0.9 and VD vs. VCIND: AUC = 0.8. However, sMRI performed poorly in distinguishing between VCIND and NC participants (AUC = 0). In the VCIND population, model features only included Putamen_L and Thalamus_R, indicating that extensive gray matter atrophy has not yet developed and structural changes in the brain are not yet evident. This is consistent with previous studies (Frantellizzi et al., 2020). The decrease in gray matter in the thalamic region among VCIND patients may be linked to a decline in executive function, as previously demonstrated by Cao et al. (2010). The VCIND group exhibited impairments in various cognitive domains, ranging from 17 to 66%, with the lowest rate in the Clock Plotting Trial and the highest in the STT-A. Additionally, there were significant reductions in regional cerebral blood flow (rCBF) bilaterally in the thalamus compared to NC. Our EEG model is comparable to previous studies in terms of classification rate (Al-Qazzaz et al., 2018; Torres-Simón et al., 2022), achieving approximately 85–90% accuracy in distinguishing between the VD and NC groups, but only 60% accuracy in distinguishing between the VCIND and NC groups. The better performance of sMRI in categorizing VD versus NC compared with resting-state EEG may demonstrate that anatomical information captured by sMRI features is more sensitive than the neurophysiological information provided by EEG. The study found that models using only EEG or sMRI features had low accuracy in distinguishing between VCIND and NC groups. However, the “composite marker” model, which combined both features, achieved a classification accuracy of 72%. This discovery could potentially lead to earlier detection and intervention in more patients with early, undetectable VCIND, ultimately reducing the growth rate of VD.

Our study combines qEEG with sMRI to build a support vector machine classification model, which not only highlights the advantages in early identification of patients with VCI, but also helps to explore biomarkers with significant differences between patients with VCI and normal subjects. The discovery of these biomarkers may help to understand the biological mechanisms of the disease and may also contribute to the search for potential therapeutic targets for VCI.

Our study has limitations. It is important to note that these limitations do not invalidate the results of our study. We did not follow the participants longitudinally, and we did not validate the predictive power of the “composite marker” model of EEG and sMRI for disease progression in patients with VCI. Future studies should expand the sample size, extract more accurate EEG and sMRI features, and conduct longitudinal studies to clarify the biological features related to the progression of patients with VCI. This will help establish a prediction model for the progression of VCI, accurately identifying and predicting the progression of patients with VCIND in the early stages when symptoms are not significant.

## Data availability statement

The raw data supporting the conclusions of this article will be made available by the authors, without undue reservation.

## Ethics statement

The studies involving humans were approved by the Medical Ethics Committee, Hongqi Hospital, Mudanjiang Medical College. The studies were conducted in accordance with the local legislation and institutional requirements. The participants provided their written informed consent to participate in this study.

## Author contributions

ZL: Conceptualization, Funding acquisition, Investigation, Project administration, Supervision, Writing – original draft, Writing – review & editing. MW: Conceptualization, Investigation, Formal analysis, Visualization, Writing – original draft. CY: Funding acquisition, Resources, Writing – review & editing. ZW: Data curation, Visualization, Writing – original draft. JW: Data curation, Writing – original draft. LC: Data curation, Visualization, Writing – original draft. WZ: Funding acquisition, Resources, Writing – original draft.

## Footnotes

1
https://sccn.ucsd.edu/eeglab/download.php

2
https://matlab.vmecum.com/

3
https://www.csie.ntu.edu.tw/∼cjlin/libsvm/
